# Sinking Skin Flap Syndrome: Phenomenon of Neurological Deterioration after Decompressive Craniectomy

**DOI:** 10.1155/2018/9805395

**Published:** 2018-10-23

**Authors:** Noman Ahmed Jang Khan, Saad Ullah, Waseem Alkilani, Hassan Zeb, Hassan Tahir, Joshan Suri

**Affiliations:** Department of Internal Medicine, Temple University, Conemaugh Memorial Medical Center, Johnstown, PA, USA

## Abstract

Sinking skin flap syndrome is rare phenomenon that occurs in patients with large craniectomies. Alteration in normal anatomy and pathophysiology can result in wide variety of symptoms including altered mental status, hemodynamic instability, and dysautonomias. Management is largely conservative. We here present a case of a patient with large craniectomy who was admitted to our hospital with pneumonia. Later on, he developed worsening mental status and CT head revealed sinking skin flap with significant midline shift. This is a very rare case of neurological deterioration after craniectomies, commonly known as sinking skin flap syndrome. To our knowledge, only few cases have been reported so far.

## 1. Introduction

Sinking skin flap syndrome, often called as the “syndrome of trephined,” is a rare complication after a large craniectomy. The procedure is thought to convert cranium from a closed to an open box, hence altering the basic pathophysiology.

Eventually, in some cases, a significant difference between atmospheric and intra cranial pressures is created. When the atmospheric pressure exceeds the intra cranial pressure, the skin flap presses on the brain tissue resulting in paradoxical herniation. This phenomenon may produce a wide range of symptoms including headache, dysautonomia, mental changes, seizures, and focal deficits. We present a rare case of an 18- year-old male who had a traumatic brain injury after a fall for which a large craniectomy and ventriculoperitoneal (VP) shunt were performed. The patient was admitted to our hospital for aspiration pneumonia and later developed neurologic deterioration. Computed tomography (CT) head revealed sinking skin flap syndrome.

## 2. Case Presentation

**Figure 1:**
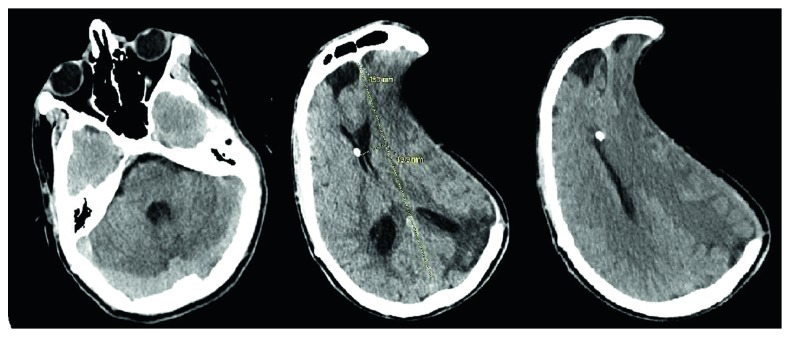
CT head showing left-sided prior craniectomy with significant midline shift.

**Figure 2:**
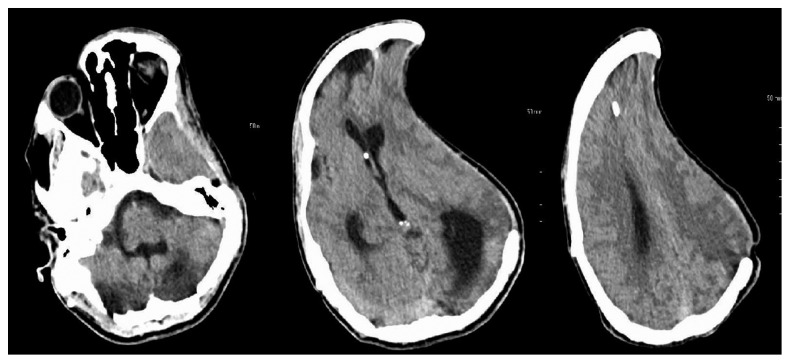
Head CT revealing left-sided prior craniectomy with improved midline shift as compared to Figure 1.

An 18-year-old male, nursing home resident, presented to the hospital with labored breathing and acute respiratory failure. The patient at baseline was nonverbal with a Glasgow coma scale (GCS) of 10/15. Approximately a year prior to presentation, the patient had suffered from traumatic brain injury after a fall. He underwent large left-sided craniectomy with a bone flap and placement of VP shunt at an outside hospital at the time. At initial presentation to our hospital, the patient was started on broad-spectrum intravenous antibiotics to cover for pneumonia. On day 3, the patient was noted to have sinus bradycardia with heart rate in the 40 s and low blood pressure at 90/60. On examination, the patient had a GCS of 8/15 with unequal pupils. Emergent CT head revealed sinking skin flap syndrome with paradoxical brain herniation and 19.9 mm midline shift (Figure 1). The patient was placed in Trendelenburg position and transferred to the intensive care unit for close monitoring. An urgent neurosurgery consult was also obtained, and cranioplasty was advised but the family deferred. The patient's VP shunt was adjusted to increase the intracranial pressure. A repeat CT scan after 5 days revealed stable midline shift with no interval changes and improvement in mental status (Figure 2).

## 3. Discussion

The syndrome of trephined is an uncommon postoperative complication that usually occurs after months of surgery in patients who undergo large craniectomy for various reasons like traumatic brain injury, malignant middle cerebral artery infarction, contusions, and subdural hematomas. The first case of sinking skin flap syndrome was reported by Yamamura et al. back in 1977 [1]. After that, sinking skin flap syndrome has been reported fairly in the literature. In a study of 108 patients performed back in 2008 who underwent decompressive crainectomy, syndrome of trephined was reported in 13% of patients between 28 and 188 days after the surgery [2].

Patients often present with symptoms of neurologic deterioration including new onset headache, dizziness, mood changes, fatigability, or seizures. Some patients present with delayed dysautonomic symptoms like postural hypotension and urinary and bowel dysfunction. Physical examination often reveals a large skull defect with an overlying depressed skin flap. The ubiquitous CT findings include paradoxical herniation, sunken skin flap sign, and deviation of the midline structures [3]. The CT head without contrast of our patient revealed small slit-like ventricles and left-sided craniectomy defect with a sunken brain/skin flap. In addition, it was noted that the adjacent brain had an outwardly concave appearance with an associated midline shift to the right by 19.9 mm (Figure 1).

The pathophysiology of this syndrome is not clear. Some authors suggest that large craniectomies convert the cranium from a closed box to an open cavity which alters the brain pathophysiology. The atmospheric pressure and gravity overwhelms the intracranial pressure which leads to paradoxical herniation and sunken skin flap syndrome [4]. Langfitt TW theorized that atmospheric pressure is directly transmitted to the intracranial cavity, which increases inward shifting of the scalp over the surgery site [5].

The treatment options in this catastrophic syndrome are limited and not clearly defined. The primary goal of treatment in syndrome of trephined is the restoration of external pressure that is exerted by the depression of the craniectomy site. The data supporting conservative management in patients with neurological deterioration are lacking. However, trendelenberg position largely reverses the paradoxical herniation in this syndrome. In some cases, intrathecal saline infusion was found to be effective in reversing the impending herniation [6].

The principal treatment option available currently is cranioplasty. It corrects the abnormal CSF dynamics by lowering the local intracranial pressure, thus improving postural blood flow and cerebral glucose metabolism. Other treatment options in patients with VP shunt include temporary occlusion or removal of shunt device before cranioplasty [7]. Our patient was managed conservatively by close monitoring in the ICU. The patient was placed in the trendlenberg position. A revision of the VP shunt was carried out by the neurosurgery. A repeat CT head revealed no interval change (Figure 2, repeat CT). Outpatient follow-up also showed no changes from the previous assessment on discharge.

## 4. Conclusion

Sinking skin flap syndrome is a catastrophic delayed complication in patients who underwent craniectomy for various reasons. It should be suspected in all patients who had skull surgery and present with new onset neurological deterioration and dysautonomic symptoms. All clinicians must be aware of this rare yet life threatening syndrome in order to avoid unnecessary testing and to prevent its occurrence.
